# Neglected volar distal radioulnar joint dislocation with an associated ulnar styloid fracture: A case report and review of literature

**DOI:** 10.1016/j.ijscr.2024.110133

**Published:** 2024-08-10

**Authors:** Raden Dadan Gardea Gandadikusumah, Gibran Tristan Alpharian, Ghuna Arioharjo Utoyo, Calvin

**Affiliations:** a Department of Orthopaedics and Traumatology, Bandung City Regional General Hospital, Bandung, Indonesia; b Department of Orthopaedics and Traumatology, Dr. Hasan Sadikin General Hospital, Bandung, Indonesia; c Bandung City Regional General Hospital, Bandung, Indonesia

**Keywords:** Volar DRUJ dislocation, Ulnar styloid fracture, Neglected case, Case report

## Abstract

**Introduction and importance:**

Volar distal radioulnar joint (DRUJ) dislocation with an isolated ulnar styloid fracture is considered as a very rare clinical entity. Due to its subtle clinical presentation, patients often presented late. Optimal management is required to prevent functional impairment and improved quality of life.

**Case presentation:**

A 51-year-old female presented to our outpatient clinic with neglected volar DRUJ dislocation and isolated ulnar styloid fracture, resulting from a previous injury that was initially misdiagnosed as a wrist sprain approximately four months prior. A plain wrist radiograph and computed tomography scan confirmed the volar DRUJ dislocation and ulnar styloid fracture without any other bony involvement. Surgical intervention was planned, and an open reduction technique was performed, consisting of Kirschner wire stabilization, volar radioulnar ligament plication, and volar capsular repair.

**Discussion:**

The involvement of the component of triangular fibrocartilage complex (TFCC) and joint capsule must be evaluated, as both of this structure plays an important role for long-term DRUJ stabilization. Repair or reconstruction must be attempted if an evidence of tears was observed intraoperatively. Temporary stabilization of the distal radioulnar joint while allowing the repaired tissue to heal can be achieved with radioulnar K-wire fixation.

**Conclusion:**

Our report suggests that this condition can be managed with a radioulnar K-wire stabilization in combination with a soft tissue repair or reconstruction. This approach was found to resulted in satisfactory clinical outcomes.

## Highlights

• Volar DRUJ dislocation with an associated ulnar styloid fracture is a very rare clinical entity.
• Involvement of TFCC and joint capsule should be address intraoperatively.
• K-wire fixation on the radioulnar was intended to temporarily stabilize and restore the radioulnar association.
• Repair or reconstruction must be performed to ensure long-term DRUJ stability if involvement of TFCC or capsule was observed.

## 1 Introduction

Volar distal radioulnar joint dislocation (DRUJ) is considered as a very rare injury pattern, accounting for about 0.02 % of all bony injuries. So far, only 36 volar DRUJ dislocation cases have been reported in the literature [1,2]. Due to the subtle clinical deformity caused by this condition, diagnosis is often easily missed, especially if it is not associated with any wrist bone fracture. More than 50 % of cases have been reported to be misdiagnosed, highlighting the importance of being vigilant in identifying this condition [3].

In the rare occurrence where the dislocation is in conjunction with a fracture, it can affect the distal part of the ulnar bone. Presently, there are only ten published reports that describe a case of volar DRUJ dislocation with an associated ulnar styloid fracture, with six being acute cases [[4], [5], [6], [7], [8], [9]] and four being neglected cases [[10], [11], [12], [13]]. Given the rarity of this condition, the management approach still varies across each report, especially for neglected cases. In addition, management of neglected condition is often challenging, as the involvement of passive or active stabilizers can significantly impact long-term clinical outcomes [14]. The involvement of this component should be addressed in order to restore the DRUJ stability. In this case report, we presented a case of neglected volar DRUJ dislocation with an associated ulnar styloid fracture, which was successfully treated surgically. This work has been reported in line with the SCARE criteria [15].

## 2 Case presentation

**Fig. 1:**
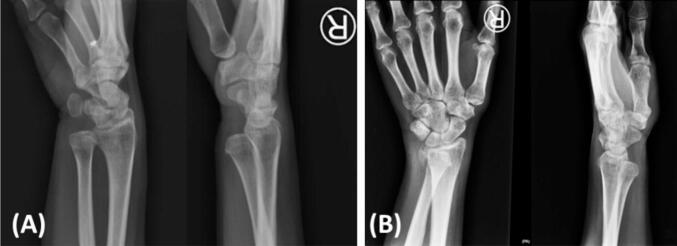
Plain radiograph after the initial injury (A) and plain radiograph at the outpatient clinic (B); both demonstrate a volar displacement of the ulna and a fracture of the ulnar styloid.

**Fig. 2:**
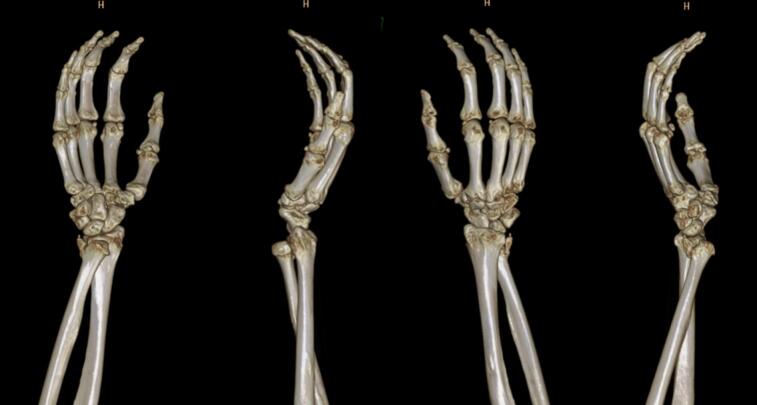
Wrist 3D CT-Scan revealed a volar DRUJ dislocation with an isolated ulnar styloid fracture.

A 51-year-old female presented to our outpatient clinic with symptoms of inability to pronate her right wrist. Approximately four months ago, the patient's wrist was accidentally struck by her granddaughter, resulting in an excessive supination force. Then, this patient visited an emergency department and was diagnosed with a wrist sprain and discharged home (Fig. 1A). The patient admitted that after this injury, her wrist was locked in the supine position. Due to the persisting symptom, the patient decided to visit our outpatient clinic. Upon our review of patient symptoms, we suspected a volar DRUJ dislocation. A plain wrist radiograph was ordered (Fig. 1B). The wrist radiograph revealed a volar DRUJ dislocation with an isolated ulnar styloid fracture. A computed tomography (CT) scan confirmed no other bony injuries (Fig. 2).

**Fig. 3:**
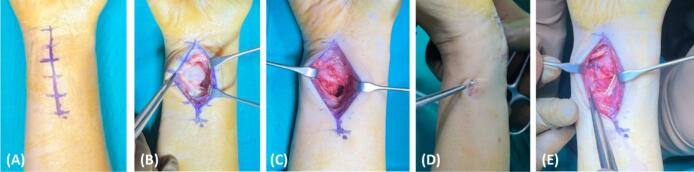
A surgical incision was made through an ulnar-volar approach to expose the DRUJ complex (A). Distal ulnar was found volarly dislocated and an abundant fibrotic tissue was seen surrounding the distal ulnar (B). The fibrotic tissue was released and removed to allow the reduction of the distal ulnar to its original position (C). A K-wire was then placed to stabilize the DRUJ complex (D). Finally, volar radioulnar ligament was plicated and volar capsule was repaired (E).

Surgical intervention was planned for this patient. Due to the chronicity of the DRUJ dislocation, closed reduction was not attempted, and we chose to manage this patient with an open reduction technique. Intraoperatively, an incision was made through the ulnar volar approach (Fig. 3A). Volar capsules were found to be torn already, and the volar radioulnar ligament was found to be laxed and elongated. The ulnar head was seen to be surrounded by an abundant amount of fibrotic tissue (Fig. 3B). Therefore, careful blunt dissection was performed. Fibrotic tissue was carefully removed to release the ulnar head. The ulnar head was then reduced back into the neutral position (Fig. 3C). Two Kirschner wires (K-wire) were inserted across the radioulnar to stabilize the DRUJ complex (Fig. 3D). The ulnar styloid was found to be already adequately attached and embedded by the soft tissue within the fracture location. The ulnar collateral ligament was also found to be intact and fully attached to the fragment of the ulnar styloid. Therefore, we do not attempt to fixate the ulnar styloid with a fixation device. Plication of the volar radioulnar ligament with 2–0 Polyglycolic Acid surgical suture (PGA, Optime®, Indonesia) was performed to tighten this ligament. The volar capsule was meticulously repaired and re-tightened using the same surgical suture (Fig. 3E). Finally, the fascia and skin were closed in the usual manner.

**Fig. 4:**
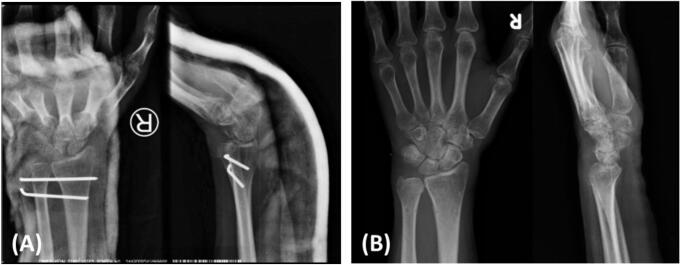
Postoperative wrist radiograph (A) revealed that DRUJ complex was held in place by the K-wire. Radiograph at 4th months (B) revealed an intact DRUJ complex and union of ulnar styloid fragment.

**Fig. 5:**
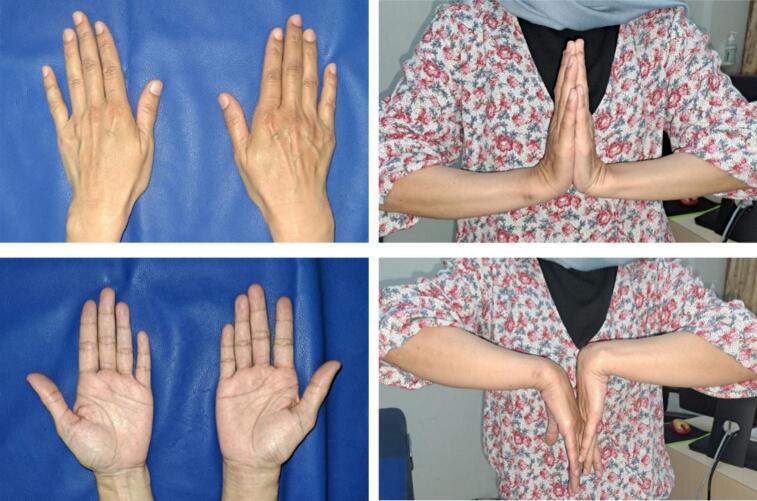
Clinical result at the final month follow-up period.

Postoperatively, the wrist was temporarily immobilized with a long arm splint (Fig. 4A). A week later, we converted it to an above-elbow cast. Approximately a month after the initial injury, the K-wire and cast were removed. The patient was then referred to a rehabilitation unit, and monthly follow-ups were conducted. At the 4th month follow-up period, the patient already reported satisfactory wrist pronation-supination motion, and the patient stated that she was able to perform her normal daily activities. Radiograph revealed a union ulnar styloid (Fig. 4B). At the 6th month follow-up, the patient stated that she almost returned to her pre-injury level (Fig. 5). Wrist flexion, extension, pronation, and supination was 70°, 90°, 90°, and 80°; respectively. Record of outcomes assessment using the Disabilities of Arm, Shoulder and Hand (DASH) score can be seen on Table 1.

**Table 1:** Outcome assessment record using the DASH score.

Follow-up period	DASH score
Pre-operative	66.7
1st month	48.3
2nd month	30.8
4th month	18.3
6th month	10.8

## 3 Discussion

No agreement has yet to be made regarding the management approach for this type of condition, with only limited reports were available so far (Table 2). Radical approach such as distal ulnar resection have been previously described by Weseley et al. [11] and Kikuchi et al. [13]. However, this approach has been associated with several complications, including: painful instability, significant strength diminution, carpus instability, risk of extensor tendon rupture, and decreased pronation-supination function [16,17]. On the other hand, a more straightforward approach has been described by Li et al. [10]. In their report, management was conducted with a single K-wire fixation on the radioulnar without any additional procedure. Unfortunately, notable wrist pronation and supination loss were still observed, with only 40° active pronation and 60° active supination.

**Table 2:** Summary of the patient demographic, duration of injury to surgery, intraoperative findings, and management of neglected volar DRUJ dislocation with an associated ulnar styloid fracture in existing literature.

Case report/case series	Year	Age/sex	Injury to surgery (months)	Intraoperative findings	Management	Follow-up
Li et al. [10]	2014	58/F	12 mo	- Dorsal radioulnar ligament rupture - Joint capsule rupture - Deformed ulnar head with damage cartilage surface - Partially attached of ulnar collateral ligament to malunited ulnar styloid	- Radioulnar fixation with single K-wire without any additional tissue reconstruction - Long arm posterior splint for six weeks	At 8 months: - Pronation: 40° - Supination: 60°
Kikuchi et al. [13]	2005	19/M	14 mo	- Malunion of ulnar styloid - Intact TFCC	- Partial resection of ulnar head - Tension band wire for the ulnar styloid fragment - Upper arm cast for two weeks	At 12 months: - Pronation: 60° - Supination: 70°
Weseley et al. [11]	1972	26/M	9 mo	- Non-union of ulnar styloid (displaced)	- Ulnar distal end resection	Not describe
Johnston et al. [12]	2009	18/F	84 mo	- Non-union ulnar styloid	- Radioulnar fixation with 3.5 mm cortical screw proximal to the DRUJ - Joint capsular plication procedure - Above elbow posterior splint for 2 weeks, followed by Munster-type cast for 8 weeks	At 26 months: - Pronation loss: -2° - Supination loss: -10° - DASH: 2.5
		23/F	36 mo	- Non-union ulnar styloid		At 19 months: - Pronation loss: -18° - Supination loss: -43° - DASH: 46.7
		36/F	18 mo	- Union ulnar styloid		At 13 months: - Pronation loss: -1° - Supination loss: -1° - DASH: 18.3
		21/F	18 mo	- Non-union ulnar styloid		At 11 months: - Pronation loss: 0° - Supination loss: -5° - DASH: 9.2

Given the complexity of the DRUJ, each anatomical structure that plays a role as a DRUJ stabilizer needs to be addressed surgically. Two crucial structures in this regard are the triangular fibrocartilage complex (TFCC) and the DRUJ capsules. Both of these structure is considered as the passive stabilizer for DRUJ [14]. TFCC function is to facilitate rotational stability, provide ligamentous support for carpus, load transmission absorbent, and ensure the continuity of the radioulnar gliding surface. Meanwhile, the DRUJ capsule acts as a resistance to the ulnar translation force [18]. Failure to recognize the important structure might result in persistent instability and unsatisfactory wrist function.

In neglected cases, direct TFCC or capsular repair sometimes might not be feasible. It occurs as a consequence of retracted remnants or due to the abundant fibrotic tissue surrounding the remnants, which makes this structure hard to be identify. In such circumstances, ligament and capsular reconstruction is necessary. As in the study of Ferreira et al. [19], reconstruction of the dorsal and volar radioulnar ligament was found to adequately preserve the pronation and supination motion in chronic instability cases. Additionally, a displaced ulnar styloid also needs to be addressed, as it often leads to distal or volar radioulnar ligament disruption. Fixation is usually mandated if there is evidence of displaced ulnar styloid fracture that is associated with TFCC ligamentous disruption [20,21].

In our case, we conducted a step-wise management approach. K-wire fixation was conducted to temporarily restore the distal radioulnar association. The laxed volar radioulnar ligament was plicated and the torn volar capsule was repaired with the aim of maintaining the integrity of the DRUJ passive stabilizers structure. This quite similar approach has been previously described in the series by Johnston et al. [12]. In their series, radioulnar screw fixation and volar capsule plication were found to be effective in the management of neglected volar DRUJ dislocation with an associated ulnar styloid fracture. The average DASH score in their patients was found to be 13 (0–46.7), with only a slight supination loss were observed. As supported in the cadaveric study conducted by Goften et al. [22], capsular repair and radioulnar ligament reconstruction were found to significantly improve and restore the kinematic of unstable DRUJ. In comparison to previous describe management approaches like the distal ulnar resection alone [11,13] or K-wire fixation alone [10], a marked loss of wrist supination and pronation function were still observed. Therefore, based on our report findings, it appears that adequate repair of involved soft tissue is needed in order to restore and match the original kinematics of the DRUJ. This approach was found to resulted in a satisfactory clinical outcomes.

## 4 Conclusion

Neglected volar DRUJ dislocation with an associated ulnar styloid fracture is considered as a very rare clinical entity. This case requires special attention, especially where there is a bony structure and soft tissue involvement. Our report indicated that radioulnar K-wire stabilization and soft tissue repair resulted in satisfactory clinical outcomes.

## Ethical approval

Case report are exempt from ethical approval according to our institution IRB.

## Funding

This case report did not receive any specific grant from funding agencies in the public, commercial, or not-for-profit sectors.

## Authors' contributions

Raden Dadan Gardea Gandadikusumah: Conceptualisation, Validation, Reviewing and Editing.

Gibran Tristan Alpharian: Conceptualisation, Validation, Reviewing and Editing.

Ghuna Arioharjo Utoyo: Conceptualisation, Validation, Reviewing and Editing.

Calvin: Methodology, Writing, Data Curation, Original draft preparation.

## Guarantor

Calvin.

## Registration of research studies

Not applicable.

## Consent

Written informed consent was obtained from the patient for publication of this study. A copy of the written consent is available for review by the Editor-in-Chief of this journal on request.

## Declaration of competing interest

Authors have no conflict of interest to declare.

## Data Availability

The data are available from the corresponding author upon reasonable request.
